# Mobilizing stakeholders for implant removals in Burkina Faso using landscape assessment data

**DOI:** 10.1186/s12905-024-03121-z

**Published:** 2024-05-20

**Authors:** Justin Tiendrebeogo, Bethany Arnold, Yacouba Ouedraogo, Rachel Haws, Lea Pepin Garane, Virginie Ouedraogo, Maria Gouem, Alimata Coulibaly, Mathieu Bougma

**Affiliations:** 1 Jhpiego Burkina Faso, Ouagadougou, Burkina Faso; 2 Jhpiego, Baltimore, MD USA; 3 Department of International Health, Johns Hopkins Bloomberg School of Public Health, Baltimore, MD USA; 4 Ministère de la Santé [Ministry of Health] Burkina Faso, Ouagadougou, Burkina Faso

**Keywords:** Family planning, Quality of care, Long-acting reversible contraceptive, West Africa, Stakeholder engagement, Stakeholder mobilization, Data for decision-making, Implanon, Nexplanon, Jadelle

## Abstract

**Background:**

Successful efforts to encourage uptake of subdermal contraceptive implants, with a lifespan of three to five years, necessitate planning to ensure that quality removal services are available when desired. In Burkina Faso, implant use has tripled over the past 8 years and now comprises almost half of the contraceptive method mix. Population Monitoring for Action (PMA) surveys identified barriers to obtaining quality removal when desired, particularly when the implant is not palpable, or providers lack needed skills or supplies. The Expanding Family Planning Choices (EFPC) project supported ministries of health in four countries with evaluation and strengthening of implant removal services.

**Methods:**

An implant removal landscape assessment was conducted at 24 health facilities in three regions of Burkina Faso with high implant use that included provider observations of implant removal, interviews with providers and health facility managers, and facility readiness surveys. The project used landscape data to mobilize stakeholders through a series of participatory workshops to develop a collaborative roadmap and commit to actions supporting quality implant removals.

**Results:**

Landscape findings revealed key gaps in provision of quality removal services, including high levels of provider confidence for implant insertion and removal (82% and 71%, respectively), low competence performing simple and difficult removals (19.2% and 11.1%, respectively), inadequate supplies and equipment (no facilities had all necessary materials for removal), lack of difficult removal management systems, and a lack of standard data collection tools for removal. Exposure to the data convinced stakeholders to focus on removals rather than expanding insertion services. While not all roadmap commitments were achieved, the process led to critical investments in quality implant removals.

**Conclusion:**

Landscape data revealed that facilities lack needed supplies and equipment, and providers lack skills needed to perform quality implant removals, limiting client reproductive choice. Disseminating this data enabled stakeholders to identify and commit to evidence-based priority actions. Stakeholders have since capitalized on program learnings and the roadmap, including following MOH guidance for implant removal supplies and health provider training. Our experience in Burkina Faso offers a replicable model of how data can direct collective action to improve quality of contraceptive implant removals.

**Supplementary Information:**

The online version contains supplementary material available at 10.1186/s12905-024-03121-z.

## Background

### The need for quality contraceptive implant removal services

In recent years, subdermal contraceptive implant use has gained popularity and has surged in use worldwide, with the highest rates of uptake in sub-Saharan African countries [1, 2]. Launched in 2013, the Implants Access Program (IAP), a public–private global collaboration, has been working to make implants accessible to women in low-income countries. Most notably, the IAP halved the price of implants globally through a volume guarantee and efforts to improve market dynamics [3]. The IAP has also worked to strengthen supply chain performance, train providers in insertion and removal, and increase community knowledge and awareness about long-acting reversible contraceptives (LARCs).

In Burkina Faso, uptake of modern contraceptive methods has climbed steadily in recent years; Performance Monitoring for Action (PMA) surveys have found that the modern contraceptive prevalence rate for women in unions increased from 18.1% in 2014 to 30.8% in 2019 [4, 5]. In part due to the volume guarantee, contraceptive implants account for half of this increase, rising from 41% of the method mix in 2014 to 50.3% in 2018, but declining to 44.1% in 2020 [4–6]. Both two-rod (Jadelle and Levoplant) and one-rod (Implanon NXT) implants are available in Burkina Faso, but more than 92% of implant users use two-rod products [6]. Burkina Faso’s national health management information system (HMIS) documents that the number of new users of implants has more than tripled from 2011 to 2020, from 55,044 to 187,290 [7, 8]. The vast majority of implants—more than 97%—are provided through public facilities [5]. Implants are also provided to clients during special family planning (FP) weeks, as well as via outreach services in both the public and private sectors.

An implant is effective for three to five years, though clients may elect removal prior to this point if they desire to become pregnant, wish to change contraceptive methods, or have any other reason for discontinuation [9]. From a rights perspective, ensuring on-demand access to quality implant removal services helps keep the promise of LARCs—that they are not only long-acting but also reversible—therefore safeguarding clients’ reproductive choice [10, 11]; access is also programmatically important as it supports continued demand for and client satisfaction with implant use, particularly in countries where contraceptive implant use has rapidly scaled up [10, 12]. However, recent global data have shown that clients do not have access to high-quality implant removal services [10, 13, 14], including in Burkina Faso, where 2018 PMA data identified an unmet need for removals of 7% [6]. While implants have grown in popularity, they remain a provider-dependent method: a client requires a provider to insert the implant to start the method and to remove the implant to stop using it, whether the method has reached the end of its effectiveness or the client wants to discontinue for any reason [10, 15].

Implants can usually be removed easily through a small opening in the skin; rarely, implants are difficult to remove because they are non-palpable, have migrated, were incorrectly inserted, or have grown encased in fibrous tissue [16]. Provider skills required for difficult contraceptive implant removals can exceed those required for standard removals. Difficult removals may require specialized provider training and equipment such as radiography and/or ultrasound when the implant is not palpable [16]. In Burkina Faso, currently only secondary and tertiary referral centers (Regional Hospital Center [CHR] and University Hospital Center) and some primary referral centers (CMA) have the required equipment to carry out removals for deeply inserted implants [17, 18]. Ultimately, the Ministry of Health (MOH) plans to extend provider training in difficult removals to all second and third level health facilities and health districts that have adequate equipment for the removal of deeply inserted contraceptive implants. This may reduce the distance a women must travel to reach a facility and provider capable of removing her implant.

### Engagement and mobilization around quality FP services in Burkina Faso

Over recent decades, the Government of Burkina Faso has committed to promote FP for the wellbeing of its population. These objectives are enshrined in the National Family Planning Acceleration Plan 2017–2020 (PNAPF) which commits to increase knowledge of and access to rights-based FP services [19]. Improving the quality of contraceptive implant removal services is an integral part of MOH efforts to systematically improve quality and supply of modern contraceptives and stimulate demand for these products.

To address the urgent need to ensure quality implant removal services, the Bill & Melinda Gates Foundation awarded Jhpiego a short-term grant through the Expanding Family Planning Choices (EFPC) Project, from May 2018 to February 2020. EFPC provided short-term technical assistance to support four countries with high implant use (Burkina Faso, the Democratic Republic of Congo, Nigeria, and Tanzania) to evaluate and strengthen quality implant removal services in alignment with each country’s family planning roadmap and in direct partnership with its ministry of health.

In Burkina Faso, we conducted a landscape review and used resulting data to engage MOH and other FP stakeholders to support quality contraceptive implant removal services. Lessons learned from this experience outline a replicable model for engagement of FP stakeholders around quality contraceptive implant removal that is readily adaptable to other contexts and sectors.

## Methods

### Project adaptation and development

**Fig. 1:**
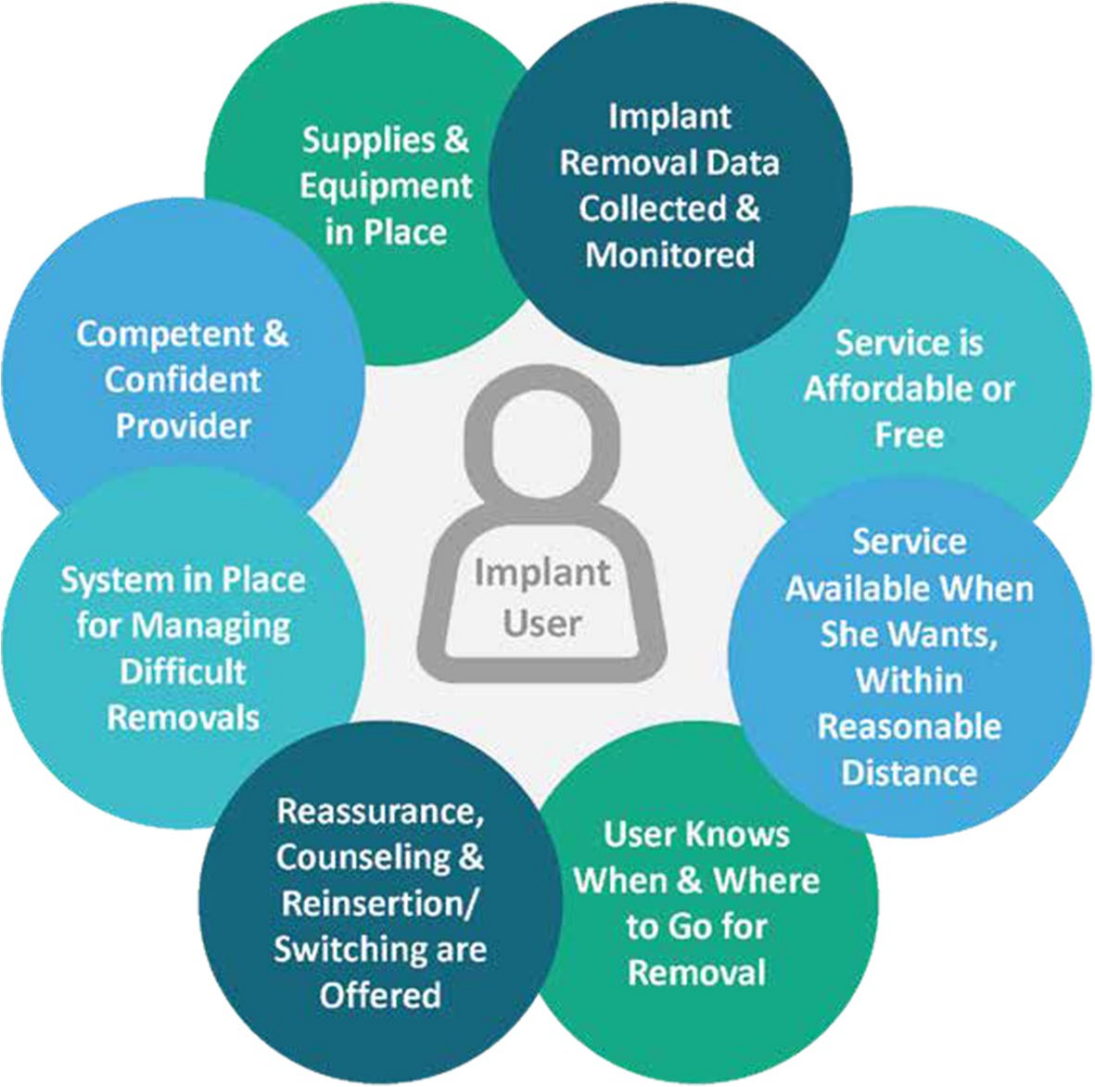
Client-centered conditions for ensuring access to quality implant removal. Source: Implant Removal Task Force of the Implants Access Program Operations Group

The EFPC project focused on eight conditions for quality implant removal services developed by the Global Implant Removal Task Force, a consortium of more than 20 key FP partners and donors, as part of the IAP Operations Group. This framework categorizes the components that must be met to ensure that a client has access to quality implant removal services [10] (Fig. 1).

A pivotal first step in engaging and mobilizing key stakeholders was to present the project to the *Direction de la Santé de la Famille* (DSF) [Directorate of Family Health], the department in charge of FP at the MOH. Acknowledging that the quality of contraceptive implant removal services merited attention, the DSF took the lead to mobilize other MOH stakeholders, including central directorates, regional directorates, and districts. The DSF organized meetings with them to present the project and encourage their involvement in the landscape assessment and other activities.

### Landscape analysis

To amass evidence to sensitize stakeholders to the need for quality removals, we conducted a landscape analysis at the inception of the project. The landscape assessment protocol was approved by the Burkina Faso Health Research Ethics Committee and by the Johns Hopkins University Institutional Review Board, which granted a non-research determination. The analysis included a desk review and primary data collection to evaluate the current status in Burkina Faso of four of the eight client-centered conditions for quality implant removal: competent and confident provider, supplies and equipment in place, implant removal data collected and monitored, and systems in place for managing difficult removals. These four conditions were prioritized based on the team’s background understanding of implant insertions and removals in Burkina Faso and feasibility of interventions to improve these conditions during the project period.

Primary data collection for the landscape analysis was descriptive and cross-sectional, combining quantitative and qualitative methods including provider observations, a facility assessment, and interviews with providers and facility managers to address four main research aims (Table 1). Three of Burkina Faso’s thirteen regions were selected to participate in the landscape analysis based on having had the highest number of implants inserted in 2017. The rationale for their selection was that areas with the most insertions will ultimately have the greatest need for removals, and these regions could thus serve as an early barometer of how well conditions for quality removals were being met. The top two health districts (*district sanitaire*) [DS] in number of implants inserted were selected from each region; within each district, the central facility in the district (hospital or CMA) and the three health facilities with the highest number of implants inserted were selected. Twenty-four health facilities were ultimately included in the landscape analysis, using the four tools described in the table below.

**Table 1:** Landscape assessment research aims and data collection tools

Research aims	Data collection tool	Description of the collection tool
Determine facility-based provider competency in implant removal in 2018.	**Tool 1:** Provider checklist for implant removal competencies (simple and difficult)	Checklist for direct observation of service providers during clinical practice of removal of implants on a client or anatomical model
Determine health facility readiness for implant removal in 2018. Identify gaps in the collection and use of family planning data, particularly for implants in 2018.	**Tool 2:** Provider interview guide	Structured questionnaire for service providers involved in family planning in health facilities
	**Tool 3:** Health facility assessment checklist	Checklist to review the physical, infrastructure, and organizational conditions for offering services related to removal of implants in the health facility
Delineate current policies, norms, and standards governing family planning, specifically the use of implants.	**Tool 4:** In-depth interview guide for facility managers	Structured questionnaire for facility managers

Data collection tools are available as Additional Files 1, 2, 3, and 4 in appendix

Informed written consent was received from all study participants, including observed providers, clients who had their implant removal conducted by a provider observed by the study team, and health facility managers. Clients who were illiterate in French were consented in their local language and provided their consent by giving their fingerprint to the consent form.

For provider observations, trained data collectors working under the supervision of a principal investigator with support from the MOH used an implant removal skills checklist (see Additional file 1) to clinically observe 35 service providers from 24 health facilities on implant removal technique in simple and difficult removal situations. The checklist was adapted from the USAID Maternal and Child Survival Program LARC Learning Resource Package module on contraceptive implants [20], and is available as Additional file 1 in appendix. Data collectors provided providers with all tools required to safely and correctly perform one simple or difficult implant removal, including modified vasectomy forceps, sterile gloves, gauze, scalpel, and a Gaumard RITA Reproductive Implant Training Arm if a real client was not available. All providers working in health and social promotion center (CSPS) or medical center (CM) maternity units were eligible to participate in clinical observations, but if more than one provider offered implant removal services at the CSPS/CM, the provider responsible for the maternity or the maternal and child health department was selected. At higher-level facilities with more providers, such as a medical center with surgical services (CMA), CM, regional hospital center (CHR), or urban CSPS, 3 providers were selected via drawing to be observed. Data collectors used inclusion and exclusion criteria to refine the selection of eligible providers. Inclusion criteria included a facility offering implant removal in the target zones, being an authorized provider for implant removal in the chosen facility, being a facility manager in a selected facility, and willingness to participate in the study.

Data collectors visited facilities during days and times when services were offered, Monday through Friday. Health facilities were aware that data collectors were coming and in some areas, providers notified clients who were in need of removals in advance. Each provider was asked to perform one simple or difficult implant removal, whether on a real client or on a RITA model, using provided materials. Simple removals were performed on clients, but if no client was present, service providers performed removals on the RITA arm. Due to a lack of available clients with difficult removals during the collection period, all deeply inserted implant removals were performed on the RITA arm by providers at level 2 facilities (CHR or CMA). The data collection team assessed providers using the checklist.

Steps assessed included counseling prior to removal, preparation for removal, removal, reinsertion, and post-removal care and counseling. The composite score on the checklist was used to rate provider mastery as good (80–100%), partial (50–79%), or non-mastery (0–49%). All observed providers were interviewed post-observation about their experiences and challenges with implant insertions and removals, including training levels, their confidence performing procedures, and interactions with clients using a structured questionnaire (see Additional file 2).

At each health facility where a facility manager was present, the facility manager was asked about facility readiness to provide implant removal services, using a facility assessment checklist adapted from the globally available Contraceptive Implant Removal: Rapid Service Readiness Assessment Tool developed by Jhpiego [21] (see Additional file 3), which documented availability and condition of materials and equipment needed for implant insertion and removal observed and functioning on the day of the visit, classified by 1) items required for insertions, 2) items required for simple and difficult removals, and 3) equipment and commodities necessary for infection prevention. Each facility manager was also interviewed using a structured interview guide (see Additional file 4) to better understand challenges and systems in place for implant removals, referrals for removals, availability of protocols and tools in health facilities, and staff training.

### Stakeholder mobilization process

Very limited funding necessitated strategic mobilization of and collaboration with stakeholders to identify and rank program priorities. In partnership with the DSF, the EFPC project first mapped key stakeholders, including Ministry of Health staff, regional and district government staff, and representatives from health facilities, civil society organizations (CSOs), non-governmental organizations (NGOs), and other technical and financial partners.

**Fig. 2:**
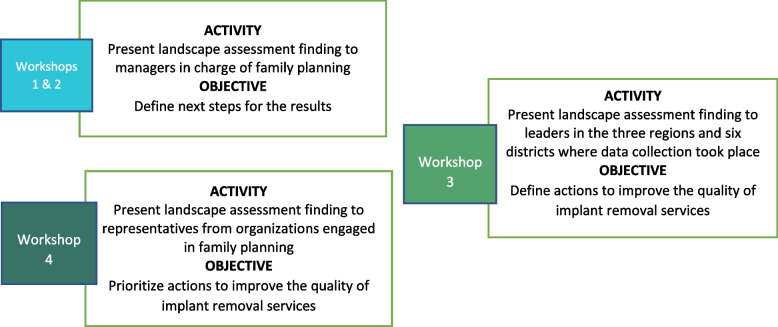
Stakeholder mobilization workshops

The project held multiple workshops to mobilize stakeholders identified through mapping (Fig. 2), including two sensitization workshops with DSF staff in January 2019, one with regional directorates and districts, and one with stakeholders from family planning organizations. At the third and fourth workshops, held in March 2019, stakeholders identified priority actions to address implant removal problems by working in small groups with an in-depth analysis of the landscape findings. Each group was asked to identify “low-hanging fruit”: actions to improve the quality of contraceptive implant removal services achievable within six months. These priority actions were used to generate a roadmap for improving contraceptive implant removal services in Burkina Faso.

Stakeholders then committed to implement roadmap activities based on their area of intervention expertise. Actions selected by each stakeholder had to be either already aligned with or easily incorporated into their current workplans. To facilitate this process, EFPC developed a commitment framework that each stakeholder used to select priority actions in the roadmap to address. After the workshop, the project held quarterly meetings with stakeholders over the following twelve months, which allowed stakeholders to share updates on roadmap implementation and progress towards their commitments. Stakeholders were subsequently encouraged to engage with other CSOs, NGOs, technical and financial partners, and other ministerial departments working in family planning and reproductive health (FP/RH). The EFPC project also engaged stakeholders from other ministries around the broader aspects of the need for quality FP services. A post-implementation workshop was held in March 2020 for representatives from the MOH, NGOs, donors, and the health system to review roadmap achievements and solicit recommendations from stakeholders to continue achievement of roadmap goals and ensure sustainability and scale-up of implant removal achievements in Burkina Faso.

## Results

### Landscape assessment results

#### Provider clinical observations

Of the 35 providers observed, 25.7% performed the removal on a client; 74.2% performed the removal on the RITA arm due to lack of clients presenting for removals during data collection. Difficult removals accounted for 28.6% of all removals (9 out of 35 removal cases) observed, and were all performed on an anatomical model due to a lack of clients presenting with difficult removals during the study period. Five of 26 (19.2%) providers observed were evaluated as competent on simple removals and 1 of 9 (11.1%) were competent on difficult removals; overall, 82.8% of providers demonstrated a lack of competence in removing implants following standard procedures. Table 2 shows provider competence by type of removal.

**Table 2:** Level of competency of service providers observed by district for each type of removal

District and Region	n	Simple implant removal (*n* = 26)			n	Difficult implant removal (*n* = 9)		
		**Poor competency**	**Partial competency**	**Good competency**		**Poor competency**	**Partial competency**	**Good competency**
DS Do	5	2	2	1	1	0	1	0
DS Dafra	4	1	2	1	1	1	0	0
***Total Hauts Bassins***	**9**	**3 (33.3%)**	**4 (44.4%)**	**2 (22.2%)**	**2**	**1 (50.0%)**	**1 (50.0)**	**0 (0%)**
DS Dedougou	4	0	3	1	2	2	0	0
DS Boromo	3	1	2	0	3	1	2	0
***Total Boucle du Mouhoun***	**7**	**1 (14.3%)**	**5 (71.4%)**	**1 (14.3%)**	**5**	**3 (60.0%)**	**2 (40.0%)**	**0 (0.0%)**
DS Leo	5	1	3	1	1	1	0	0
DS Sapouy	5	1	3	1	1	0	0	1
***Total Centre-Ouest***	**10**	**2 (20.0%)**	**6 (60.0%)**	**2 (20.0%)**	**2**	**1 (50.0%)**	**0 (0.0%)**	**1 (50.0%)**
***Total***	**26**	**6 (23.1%)**	**15 (57.7%)**	**5 (19.2%)**	**9**	**5 (55.6%)**	**3 (33.3%)**	**1 (11.1%)**

Table 3 summarizes provider competence using the procedure checklist for simple and difficult removals. Providers had the highest mastery for pre-removal tasks (e.g., handwashing, antiseptic skin preparation), the removal itself, and pre-removal counseling (e.g., welcoming the client, describing the procedure) and lowest scores in post-removal counseling (e.g., follow-up steps, counseling for another contraceptive method if desired), and getting ready (e.g., ensuring tools are ready, palpating the implant rod).

**Table 3:** Percentage of service providers with a good mastery of simple and difficult removal steps by region and district

Removal steps	Boucle du Mouhoun Region		Centre-Ouest Region		Hauts-Bassins Region		Total
	**DS Boromo**	**DS Dedougou**	**DS Leo**	**DS Sapouy**	**DS Dafra**	**DS Do**	***n* = 35**
	***n* = 6**	***n* = 6**	***n* = 6**	***n* = 6**	***n* = 5**	***n* = 6**	
Pre-removal counselling	3	2	1	2	1	2	**11 (31.4%)**
Getting ready	0	1	0	1	1	3	**6 (17.1%)**
Pre-removal tasks	2	3	3	4	4	5	**21 (60.0%)**
Removal	1	3	4	4	2	1	**15 (42.9%)**
Post-Removal tasks	2	1	2	2	0	0	**7 (20.0%)**
Post-Removal counselling	0	0	2	2	0	0	**4 (11.4%)**

#### Provider interviews

Table 4 highlights reported confidence of observed service providers for implant insertion and removal. Approximately 83% of service providers said they were confident or very confident in implant insertion, and 71% said they were confident or very confident in implant removal.

**Table 4:** Confidence of observed service providers by region

Experience	Boucle du Mouhoun Region	Centre-Ouest Region	Hauts-Bassins Region	Total
	***n* = 12**	***n* = 12**	***n* = 11**	***n* = 35**
**Provider confidence: implant insertion**				
Not confident	0	0	0	**0 (0%)**
Somewhat confident	0	0	1	**1 (2.8%)**
Moderately confident	0	5	0	**5 (14.2%)**
Confident	9	6	8	**23 (65.7%)**
Very confident	3	1	2	**6 (17.1%)**
**Provider confidence: implant removal**				
Not confident	0	0	1	**1 (2.8%)**
Somewhat confident	0	0	0	**0 (0%)**
Moderately confident	2	7	0	**9 (25.7%)**
Confident	9	4	8	**21 (60.0%)**
Very confident	1	1	2	**4 (11.4%)**

In interviews, 37% of service providers stated that they had witnessed situations where a client requesting implant removal did not receive it. The majority of service providers who experienced difficulties related to implant removal or with using removal instruments and equipment mentioned deep insertion (65.7%) and defective or missing forceps (71.4%) as the main challenges. Table 5 below provides additional details.

**Table 5:** Service providers who have experienced difficulties in implant removal services

**Difficulty**	Boucle du Mouhoun Region		Centre-Ouest Region		Hauts-Bassins Region		**Total**
	**DS Boromo**	**DS Dedougou**	**DS Leo**	**DS Sapouy**	**DS Dafra**	**DS Do**	
	***n* = 6**	***n* = 6**	***n* = 6**	***n* = 6**	***n* = 5**	***n* = 6**	***n* = 35**
**Service providers who experienced difficulty in removing implants**							
Deep Insertion	5	3	4	5	3	3	**23 (65.7%)**
Vaginal bleeding	1	0	0	0	0	0	**1 (2.8%)**
Missing rods	1	0	1	0	0	0	**2 (5.7%)**
Lack or inadequacy of instruments/equipment	0	3	1	3	1	2	**10 (28.5%)**
Lack or insufficiency of consumables	0	1	0	0	0	0	**1 (2.8%)**
Heavy workload (unavailability)	0	0	0	0	0	0	**0 (0%)**
Client who is unable to pay the cost for removal	0	0	0	0	0	0	**0 (0%)**
Lack of a competent service provider	0	0	0	0	0	0	**0 (0%)**
Other	1	0	2	0	0	0	**3 (8.5)**
**Provider having experienced difficulties in using equipment necessary for removal of implants**							
Power outage/blackout	0	1	0	0	0	0	**1 (2.8%)**
Defective or missing forceps	4	5	4	4	3	5	**25 (71.4%)**
Defective autoclave	0	0	0	1	0	0	**1 (2.8%)**
Defective sterilizer	0	1	0	0	0	1	**2 (5.7%)**
Other	1	1	1	2	2	1	**8 (22.8%)**
No difficulty	1	1	2	0	1	0	**5 (14.2%)**

Providers and managers indicated that quality of removal services was limited or jeopardized by a lack of necessary protocols, materials, and equipment, including a lack of infection prevention and control materials, and materials such as modified vasectomy forceps or curved and straight mosquito forceps required for removals.

#### Facility assessment

Table 6 highlights that no facility had the necessary equipment for insertion and removal, while 75% had equipment and supplies for infection prevention.

**Table 6:** Number of health facilities with materials and equipment per type of implant service by region

Materials and equipment per type of implant	Boucle du Mouhoun Region (*n* = 12)	Centre-Ouest Region (*n* = 12)	Hauts-Bassins Region (*n* = 11)	Total
				***n* = 24**
Health facility with all the necessary equipment for simple and difficult insertion and removal	**0**	**0**	**0**	**0 (0.0%)**
Health facility with equipment for the prevention of infections	**6**	**5**	**7**	**22 (91.7%)**

Seventeen percent and 13% of facilities had an ultrasound or X-ray machine, respectively. Eight percent had a functional autoclave, 46% had a functional sterilizer, and 25% had a pressure cooker, raising concerns about the sterilization of materials. No facilities (0 of 24) had appropriate forceps for removal (modified vasectomy forceps or the curved and straight mosquito forceps). While implant insertion and removal services were available at all health facilities included in the landscape assessment and offered 7 days per week (except at facilities in Hauts-Bassins, where services were available 5 days per week), few facilities had guidelines on implant removal. 16.6% of facilities (4 of 24) reported having guidelines, checklists, or other learning materials on hand for implant removal. Those that reported having these guidelines pointed to posters, checklists, or training materials from district-level trainings**.** Table 7 describes the presence of available supplies for simple and difficult removals, as well as infection prevention, in study facilities by region and health district.

**Table 7:** Availability of materials and equipment for implant services by region and district

Rate of availability	Boucle du Mouhoun Region		Centre-Ouest Region		Hauts-Bassins Region		Total *n*	Total % *n*=24
	**DS Boromo (*n* = 4)**	**DS Dedougou (*n* = 4)**	**DS Leo (*n* = 4)**	**DS Sapouy (*n* = 4)**	**DS Dafra (*n* = 4)**	**DS Do (*n* = 4)**		
**Implant insertion/removal (simple)**								
Kidney dishes	3	2	4	4	3	1	17	**70.8%**
Gallipot	3	3	4	4	2	2	18	**75.0%**
Mosquito artery forceps – straight	4	4	3	1	3	3	18	**75.0%**
Mosquito artery forceps – curved	3	4	2	4	4	3	20	**83.3%**
Modified vasectomy forceps	2	1	3	2	1	1	10	**41.6%**
Scalpel blades	2	3	4	4	3	4	20	**83.3%**
Scalpel holder	2	2	3	3	3	3	16	**66.6%**
Surface/field for equipment	1	0	0	0	0	0	1	**4.1%**
Lidocaine without epinephrine 1%	3	4	4	4	4	4	23	**95.8%**
5 cubic cm syringe	3	4	4	4	4	4	23	**95.8%**
Injectable water	4	4	4	3	3	4	22	**91.6%**
Sterile compresses	3	4	4	4	3	3	21	**87.5%**
Sterile bandage	4	4	4	4	3	4	23	**95.8%**
Sterile gloves	3	4	3	4	3	4	21	**87.5%**
Povidone iodine	4	4	4	4	3	4	23	**95.8%**
Armrest table	0	0	4	4	2	1	11	**45.8%**
**Total with all available and adequate insertion/removal materials**	**0**	**0**	**0**	**0**	**0**	**0**	0	**0.0%**
**Removals (difficult)**								
Ultrasound	1	1	1	1	1	0	5	**16.6%**
Radiography/x-ray machine	1	1	1	0	0	0	3	**12.5%**
**Total with appropriate additional equipment for difficult removals**	**1**	**1**	**1**	**0**	**0**	**0**	3	**12.5%**
**Other equipment**								
Functional autoclave	0	1	1	0	0	0	2	**8.3%**
Functional sterilizer ("poupinel")	3	1	2	1	2	2	11	**45.8%**
Pressure cooker	0	0	1	3	2	0	6	**25.0%**
Gynecological table	4	4	3	4	2	2	19	**79.1%**
Light source	4	1	2	4	4	2	17	**70.8%**
**Total with all other available and appropriate equipment**	**0**	**0**	**0**	**0**	**0**	**0**	0	**0.0%**
**Infection prevention**								
Running water	3	3	3	2	3	4	18	**75.0%**
Decontamination receptacles	4	3	4	4	4	4	23	**95.8%**
Safety boxes	4	3	4	4	4	3	22	**91.6%**
Soap	3	3	4	4	3	4	21	**87.5%**
Chlorinated water	4	3	4	4	4	4	23	**95.8%**
**Total with all available and appropriate infection prevention materials**	**3**	**3**	**3**	**2**	**3**	**4**	18	**75.0%**

#### Manager interviews

While data collectors tried to meet with decision-makers or managers at all study facilities (*n* = 24), decision-makers or managers were only available at 21 facilities when data collectors conducted their visits. Difficulties managers reported included provider lack of competency, lack or inadequacy of materials/equipment, lack of specific data collection methods for implant removals, and lack of communication between providers and clients on availability of implant insertion and removal services. No facility had a specific system in place to manage difficult implant removal cases; managers reported that they are handled on a case-by-case basis and referrals are generally made to higher level facilities, believing these facilities have more competent providers and materials and equipment needed to perform difficult removals.

Managers identified issues tracking implant removal data, as implant removal data were not routinely collected in the usual data collection system, whether for use at the facility level or at the district or national levels. All health facilities identified difficulties in collecting and managing data due to the lack of suitable tools for data collection, leading to non-reporting or under-reporting of removals. Managers noted that those who report removal cases use FP documents (client’s logbook, notebook, or file), but these documents capture no standard information on implant removals. Some reported using the area documenting method change to record removals. Some managers noted that removals performed during shifts (generally 12:00 a.m.-3:00 p.m. or 5:00 p.m.—7:00 a.m.) may not be recorded because of provider workload during shifts.

### Stakeholder Mobilization Results

Stakeholder mapping captured a total of 33 institutions/organizations who were invited to participate in the series of stakeholder workshops, where they were sensitized to concerns about the quality of contraceptive implant removal services revealed by the landscape assessment. Stakeholders were initially resistant to the need to focus on implant removals, believing that quality implant removal was not a large problem, that providers already had the requisite removal skills, and that the focus should be on implant insertions rather than removals. Highlighting gaps identified in the landscape assessment was a key strategy to raise awareness among stakeholders and to define priorities for action to improve the quality of removal services.

At the third and fourth workshops, a total of 15 priority actions, each of which was linked to a client-centered condition for contraceptive implant service delivery, were identified based on the landscape analysis findings and included in the roadmap. Feedback shared during these meetings indicated that the dissemination of the landscape assessment findings had alerted stakeholders to shortcomings in the quality of contraceptive implant removal services. Mirroring the breadth of the desk review, the workshop working group expanded the roadmap beyond the initial four priority client-centered conditions. After the roadmap was designed, implementing partners committed to priority actions. An example of a completed stakeholder commitment framework is presented in Table 8.

**Table 8:** Example of Framework for Stakeholder Commitment to the Implant Removal Quality Improvement Roadmap

Institution/project name	Commitment/Interest:			
	**1. What are you already doing to reduce and resolve the problems associated with implant removal?**	**2. What can you do *now* in your activities to address current challenges faced in implant removal? (Short term)**	**3. What adaptations could you make to your programs to avoid problems with implant removal in the future? (Long term)**	**4. At what level of the roadmap do you want to be involved with regard to improving access to quality implant removal services?**
**[Implementing partner NGO]**	Implant removals are part of our organization’s FP activity package	Service providers will continue to perform implant removals at the request of clients	Use the NGO’s digital mobile outreach tools to reinforce the skills of service providers in the field	Priority action 3: Point C: Use the NGO’s digital mobile outreach tools to build provider skills in the field Point E: Provide coaching and mentoring Point F: Conduct periodic assessments of service providers’ skills during supportive supervision

Outcomes of the EFPC project, including stakeholder contributions, are presented below by roadmap priority action (Table 9).

**Table 9:** Priority Actions, Activities, and Outcomes of the Quality Implant Removal Roadmap

Priority Action	Activity	Outcomes
**Competent & confident service provider**		
01 Strengthen clinical practice in pre-service training schools (National School of Public Health and other health schools)	a) Advocate for a revision of the hourly load in skills laboratories and the reinforcement of clinical supervision in training schools	21 instructors from 7 public pre-service training schools for midwives and birth attendants were trained to teach implant insertion and removal to ensure that graduates from these school are competent providers who understand the importance of high-quality implant removal services
02 Ensure the application of existing standards, norms, and protocols	a) Ensure the replication and dissemination of standards, norms, and protocols for implant insertion and removal at all levels of the health system	Used a participatory approach, led by the DSF, to adapt globally-available training modules and tools on simple and difficult removals to the Burkina Faso context and in line with updated standards, norms, and protocols for use in clinical trainings and client care nationally
03 Strengthen providers' skills by focusing on on-site FP/RH training	a) Identify clinical FP training needs of regions and districts during supervision of health facilities	New modules on standard and difficult implant removals from the adapted training curricula were integrated into the national facility-based training package
	b) Advocate for necessary resources from technical and financial partners to train providers	No action
	c) Use the digital mobile outreach tools to build provider skills in the field	No action
	d) Plan and conduct training sessions for FP service providers at health facilities	Revised training modules were used for facility-based FP training sessions for training of trainers using anatomical models. Trainers then cascaded their learning to providers at their own facilities. This approach was used by EFPC and three other stakeholders to train a total of 428 service providers from 27 health districts during the project period, and were later expanded to all districts
	e) Provide coaching and mentoring	No action
	f) Conduct periodic assessments of service providers' skills during supportive supervision	No action
**System in place for managing difficult removals**		
04 Strengthening the competence of service providers in difficult implant removal	a) Train trainers in routine and difficult implant removal	Stakeholders trained 10 service providers from the Centre and Centre-Ouest regions on the guided removal of contraceptive implants and deeply inserted implants for managing difficult removal cases in the referral centers. Trained providers developed an operational action plan to guide further activities in the field
	b) Adapt memory aids for difficult removals	Adapted globally available job aids for standard removal [22] and deeply inserted removal [23] for the Burkina Faso context
	c) Adapt the difficult removal algorithm	Translated the global difficult removal algorithm into French [24] and adapted it for the Burkina Faso context
	d) Provide health facilities with reference manuals on difficult removals and the management of side effects	Copies of the *Manual for Localizing Deeply Placed Contraceptive Implants with Ultrasound Assistance* (*N* = 1950 copies) were distributed to the DSF to be given to stakeholders and projects planning to train providers in health facilities
**Supplies & equipment in place**		
05 Acquire equipment and medico-technical material for health facilities	a) Advocate with technical and financial partners for provision of medical and technical equipment to health facilities (removal and insertion kits, infection prevention supplies, etc.)	Implant removal kits and infection prevention supplies and equipment were provided to all training sites as defined by quality standards. EFPC used a SMART Advocacy approach with the MOH to ensure appropriate supplies and equipment for implant removals at health facilities. The Secretary General for Health signed a technical note which was disseminated to all FP implementing partners indicating required components of implant removal kits (i.e., a “standardized list of materials”) and obliging them to follow the guidance. Nearly 900 kits were distributed to 171 health facilities during project implementation period by 4 stakeholder-managed projects
	b) Acquire medical and technical equipment for insertion and removal of LARCs in health facilities as per their needs	
	c) Distribute medical equipment for the insertion and removal of LARCs acquired for health facilities as per their needs	
	d) Follow up on management and handling of the distributed equipment in the field	
06 Provide health facilities with quality medical supplies and equipment	a) Provide supplies and equipment for insertion and removal of LARCs to health facilities used for training	
07 Strengthen the mechanism for the management and monitoring of supplies and equipment for LARC insertion and removal	a) Update tools for the management and periodic monitoring of materials and supplies for LARC insertion and removal	
	b) Inventory medical and technical equipment for the insertion and removal of LARCs available in health facilities	
	c) Conduct periodic inventory of materials and supplies for LARC insertion and removal	
08 Ensure the dissemination of the new Infection Prevention and Control guidelines	a) Make the new guidelines available in health facilities and the Health Education and Promotion Department	The MOH is committed to making the national infection prevention and control guidelines and other FP guidelines pertinent to implant removals widely available to health workers at health facilities. EFPC ensured that implant removal services followed the national Burkina Faso guidelines around healthcare infection prevention and control [25]
09 Make available technical guidelines, communication, and data collection tools at health facilities	a) Assess the need for technical guidelines as well as communication and data collection tools	No action
	b) Adapt simple and user-friendly tools for service providers (checklists, job aids, posters, etc.)	No action
	c) Provide health facilities with technical guidelines as well as communication and data collection tools approved by the MOH	No action
**Implant removal data collected & monitored**		
10 Consider implant removal indicators when revising data collection tools	a) Advocate with the Health Sectoral Statistics Department for the integration of implant removal related indicators during the revision of collection tools (FP Register, etc.)	Adding new indicators to the HMIS is challenging and often contentious. The EFPC project led advocacy efforts with the MOH and other stakeholders to include indicators on contraceptive implant removals in the HMIS. As a result, two indicators related to implant removal—cases of implant removal (in the FP registry) and reason for removal (on the FP card)— have been included in a 2021 review of data management tools Collecting this data will allow health workers to conduct regular reviews at facilities to act to improve the quality of removals. Removal indicators will not yet be included in the national reporting forms but will be available at the facility level. Further efforts must promote effective use of this data at facility level by facility leadership, district managers, and other stakeholders, and analysis of aggregated data for effective decision making at regional and national levels to improve services and identify problems with data quality
11 Include the removal of LARCs on the agenda of semiannual meetings held by reproductive health stakeholders	a) Define indicators related to the removal of LARCs	These priority actions focused on the Regional and District levels. Advocacy and analysis around implant removal indicators continues to raise awareness of local stakeholders about the need to ensure better quality implant removals
	b) Include LARC indicators on the agenda of semiannual meetings held by reproductive health stakeholders	
	c) Analyze and monitor indicators related to the removal of LARCs according to the framework	
12 Conduct implementation research on the reasons for implant removals	a) Include implementation research on the reasons for implant removals in district and regional action plans	No concrete achievements on action 12 (implementation research) during the implementation period. With the inclusion of contraceptive implant removal data in the FP registry, moving forward, conducting implementation research should be more feasible at the level of health facilities
	b) Conduct surveys to understand clients' reasons for implant removal	
**Services available when she wants, within reasonable distance**		
13 Strengthen communication for SBC	a) Integrate information on implant removal into the community outreach package	These priority actions focused on the Regional and District levels. The department in charge of community health workers planned to lead this by developing an adapted communication tool to be used by the community health workers for sensitization sessions in communities. Unfortunately, this priority action could not be completed due to lack of financial resources
	b) Develop key messages on implant removal in family planning counseling materials to ensure that clients know when and where to go for services	No action
**Service is affordable or free**		
14 Ensure that family planning services are free	a) Ensure that accredited NGOs take into account the free FP policy in health facilities during their facility and community visits	Implementation of the project coincided with the introduction of the free FP policy in Burkina Faso, which includes implant removal, eliminating client costs. Should clients be charged for removals, the MOH has vowed to ensure they are reimbursed, and prevent providers and facilities from continuing to charge clients these costs
	b) Monitor results produced by supervisory NGOs	No action
	c) Carry out joint monitoring of implementation of free FP services	No action
**User knows when & where to go for removal**		
15 Create demand for family planning services and information on the availability of implant removal services	a) Train and equip community health workers for community-based activities, including counseling and outreach activities	As part of roadmap implementation in intervention districts and through special FP campaigns, stakeholders engaged in demand creation through raising awareness of the availability of removal services

At the post-implementation workshop, stakeholders made several recommendations, detailed in Table 10.

**Table 10:** Stakeholder Recommendations

Recommendation	Rationale
1. Increase the number of service providers skilled in contraceptive implant removal by using appropriate training tools and a pool of trainers to conduct on-site training at the district level	Training tools developed by all stakeholders during the implementation of the roadmap under the leadership of the MOH should serve as references for the training of service providers in health facilities
2. Make contraceptive implant removal materials available at the health facility level by referring to the standardized list of removal materials for orders and supplies	Facility, district, and regional inventory management systems, including centralized mapping of materials and equipment, are required to ensure consistent availability of supplies and equipment for removals in line with demand and staffing. Regular inventories are needed to ensure that facilities are following established stock management procedures for standardized infection prevention materials and implant removal kits
3. Ensure that a system is in place for the referral of difficult cases to medical centers with surgical units and hospitals where there are trained personnel to manage them	The MOH must lead implementation of this recommendation to ensure that clients reach facilities with ultrasound and trained staff capable of performing difficult (deeply inserted or migrated) removals. Implementation would reduce risky removal attempts that often end in failure, discouraging clients. During the project, a pool of 10 providers from 3 hospitals and 2 CMAs were trained in difficult removal; this training should be scaled to all hospitals and CMAs
4. Use routine data on contraceptive implant removals for decision making at all levels of the health system to continuously improve the quality of contraceptive implant removal services	Data on implant removal is as important as that for insertion and is needed to track removals relative to insertions. Its availability at facility, district, regional, and national level is critical for evidence-based decision-making regarding access to implant removal services

## Discussion

Most research to date on contraceptive implant removal services in low- and middle-income countries has emerged from a handful of countries and has focused primarily on issues of client access, client satisfaction, and the feasibility and safety of strategies like task-shifting to community health workers to accelerate scale-up of implant removal services in low- and middle-income countries [26–30]. Few studies have assessed the quality of implant insertion or removal services, even as implants skyrocket in popularity. Our landscape assessment identified a disjuncture between self-reported provider confidence in performing removal and checklist-assessed competence performing removal under observation. The limited evidence on provider confidence and competence in implant removal is mixed. Cross-sectional studies in Senegal found that more than 90% of providers trained in implant removal felt confident in providing removal services and clients were generally positive about their access and removal experiences [27, 28].

While all study facilities reported performing removals, we observed a widespread lack of globally recommended equipment and supplies for performing simple and difficult removals, as well as infection prevention materials. Both managers and providers were concerned about the quality of removals performed without adequate equipment and supplies. Providers in Senegal also reported experiencing shortages of equipment and supplies, as well as challenges when performing difficult removals; while 72% had adequate equipment and supplies for simple removals, only 8% could manage difficult removals [27]. However, an EFPC landscape assessment in Nigeria found that 70% of providers struggled to perform implant removals, particularly in cases of deeply inserted implants [31]. Most providers in the Nigeria study lacked knowledge of implant removal steps and reported low confidence in performing removals; no facilities had all equipment and supplies required for implant removal. Providers would benefit from improved checklist-based training, job aids, and supervision; facility readiness could be improved with policies that support effective procurement of the globally recommended standardized list of required supplies and equipment.

Our landscape assessment also highlighted the need to promote development and use of indicators to track implant removals, as well as systems to manage difficult removals, including effective referrals. Even though data on removals can highlight quality of care issues with contraceptive implants, global data on the number of LARC removals is scant because few countries track this indicator. Even more rarely documented are reasons for removal, incidence of difficult removals, complications of removals, and implant discontinuation or switching contraceptive methods [32]. Multiple studies have investigated reasons for and rates of implant discontinuation, but there has been little attention to data collection on implant removals [33, 34]. However, a recent pilot study of providers in Mozambique used a supportive supervision checklist to assess the feasibility and usefulness of tracking five removal indicators recommended by the Implants Access Program task force [32]. Providers were supportive of the indicators, leading the study to recommend inclusion of removal outcome, reason for removal, and duration of use in the HMIS. In response to study findings, the Mozambique MOH has revised the FP register book and is working to integrate the three new indicators in the HMIS. Regarding systems for managing difficult referrals, despite recent recognition that having a referral system in place is essential for provision of quality implant removals [10, 14], there are no studies of referral protocols or pathways for deeply inserted or migrated implants in low- and middle-income countries (LMIC). In higher-resource countries, referral clinics and specialists including plastic surgeons may receive referrals for difficult removals [16], but in LMICs these options may be infeasible, with clients better served by improving local capabilities to perform difficult removal and/or systems that ensure clear and effective referrals to facilities with trained providers, operating theaters, and/or appropriate ultrasonographic or X-ray equipment [31].

At the end of the EFPC project, several other major projects in Burkina Faso were also supporting FP/RH programs. Some have capitalized on program learnings and the stakeholder roadmap, including following MOH requirements pertaining to equipment for removals and ensuring implant removal is integrated into training for health providers. Ongoing monitoring by stakeholders involved in roadmap implementation, with leadership by the MOH, would reveal the extent to which implant removal services are included in these organizations' service delivery programming, capacity building, and future achievements in service of completion of the roadmap. Over the course of the EFPC project, unmet removal need in Burkina Faso as measured in PMA surveys dropped from 7% in 2018 to 3% in 2020, suggesting that national investment in implant removal services, collaborative efforts to engage stakeholders, and other FP/RH programs may be bearing fruit [5, 6].

**Fig. 3:**
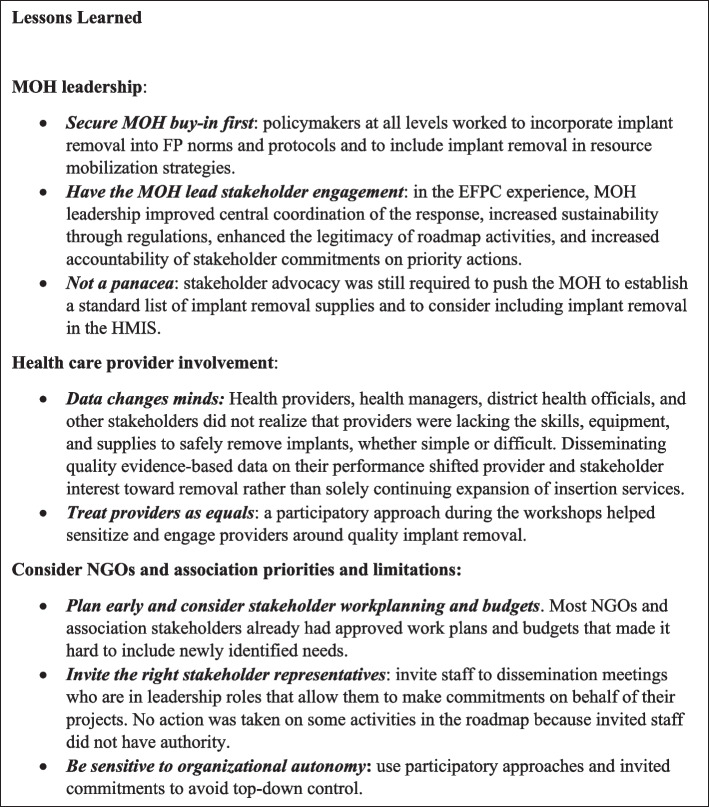
Lessons learned

A number of lessons emerged from this landscape analysis and stakeholder engagement experience (Fig. 3). Improving quality of care requires political will and leadership from the MOH and sustained stakeholder engagement, especially for family planning, as improved access to care does not guarantee quality of care needed to improve maternal health outcomes [35]. The MOH is central to prioritization, leadership, planning, and financing of any sustainable quality improvement activity, and should be involved during the planning phase of a project or intervention [36]. Establishing a culture of stakeholder engagement—or capably navigating the existing culture—requires careful mapping to ensure inclusion of stakeholders (and specific representatives) with high interest and high influence [37]. Additionally, clear communication of priorities and involving stakeholders in consultations at early stages can avoid tokenistic involvement or problems resulting from involving stakeholders too late in the process [37–40]. When working to secure stakeholder commitments, it is crucial to be sensitive to local organizations that are often most actively involved in providing care, but may concerned about top-down influences over their activities [37, 41]. In a systematic review of interventions in sub-Saharan Africa, local ownership and mobilization were frequently identified by included studies as crucial to sustainability of interventions, both early on and after intervention implementation [42].

**Fig. 4:**
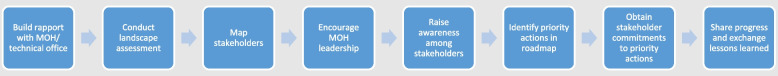
Steps in stakeholder mobilization for data-driven health roadmap development

The stakeholder engagement and mobilization approach used in this project could prove useful in other contexts and for issues beyond contraceptive implant removal. A summary of steps for effective stakeholder engagement and mobilization is shown in Fig. 4.

These ordered steps echo aspects of phases detailed in other emerging models of stakeholder engagement for quality improvement programs and policies, which include situation analyses, mapping, establishing governance, and development of plans for continuous communication and advocacy [35, 43–47]. Amassing and disseminating data revealing poor facility readiness and low provider competence in conjunction with early engagement of key policy players at the MOH fueled FP stakeholder buy-in during the workshops; participatory approaches ensured inclusion of all stakeholders and motivated them to commit to roadmap actions. Supporting development of a culture for data demand and use, including use for advocacy purposes, encourages timely and accurate data collection, leading to improved design of FP interventions in target populations [41]. Following a model of data-driven stakeholder engagement like this one can promote more systematic and meaningful stakeholder involvement, and integration of contributions by key players into a broader strategy for quality improvement.

## Conclusion

Driven by dissemination of quantitative and qualitative data that revealed low provider skill and confidence in performing implant removals, as well as poor facility readiness, a coordinated and participatory approach to stakeholder mobilization led by the MOH in Burkina Faso solicited partner contributions and capitalized on organizational strengths. Collecting and presenting data from the landscape assessment to stakeholders at the outset of the mobilization effort addressed misconceptions and sensitized them to the importance of the need for quality implant removal; encouraging MOH leadership enhanced ownership and sustainability of the stakeholder mobilization approach. Use of a roadmap allowed visual assessment of progress toward goals and increased accountability of stakeholders to honor their commitments to achieve priority actions to ensure that quality implant removal is available when desired for all users in Burkina Faso. Only if these commitments are honored by stakeholders will the promise of LARCs—that they are reversible on demand—be realized. The steps followed in this stakeholder mobilization effort form a replicable model for data-driven stakeholder mobilization that is readily transferable to other sectors and projects.

### Supplementary Information

**Supplementary Material 1. **

**Supplementary Material 2. **

**Supplementary Material 3. **

**Supplementary Material 4. **

## Data Availability

The tools used for the landscape assessment as well as datasets generated and/or analyzed during the current study are available in the FigShare repository at 10.6084/m9.figshare.23705772.v1. Repository: Figshare. This project contains the following underlying data: • Tools: 10.6084/m9.figshare.23705772.v1 • Codebook: 10.6084/m9.figshare.23705769.v1 • Datasets: 10.6084/m9.figshare.23704026.v1 Data are available under the terms of the Creative Commons CC by 4.0 license.

## Abbreviations

CHR: Regional hospital center
CM: Medical center
CMA: Medical center with surgical services/primary referral center
CSO: Civil society organization
CSPS: Health and social promotion center
DS: Health district
DSF: Directorate of Family Health
EFPC: Expanding Family Planning Choices
FP: Family planning
FP/RH: Family planning and reproductive health
HMIS: Health management information system
IAP: Implants Access Program
LARC: Long-acting reversible contraceptive
LMIC: Low- and middle-income country
MOH: Ministry of Health
NGO: Nongovernmental organization
PMA: Performance Monitoring for Action
RH: Reproductive health
