# Supplementary material for: Mobilizing stakeholders for implant removals in Burkina Faso using landscape assessment data
Source: BMC Womens Health. 2024 May 20;24:301. doi: 10.1186/s12905-024-03121-z (PMC11104007; doi:10.1186/s12905-024-03121-z)
Supplement: Supplementary file 1 — Supplementary Material 1. [file 12905_2024_3121_MOESM1_ESM.docx]

**Principal investigator:** Yacouba Ouedraogo

**Study title:** Situation analysis on the availability and quality of contraceptive implant removal services in Burkina Faso in 2018.

**Date:** September 20, 2018

**Tool 1: Checklist: Clinical skills for contraceptive implant removal**

| **Evaluator:** Use this tool when the provider performs contraceptive implant (hereinafter: implant) removal to assess his/her competence in this skill**.**  **Check 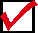** the box if the task/activity is performed **satisfactorily,** and/or 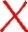 if it **has not** been performed **satisfactorily, N/O** if it has not been observed or **N/A** if the task does not fit the case observed**.**   - **Satisfactory:** Performs step or task according to standard procedure or guidelines**.** - **Unsatisfactory:** Does not perform the step or task according to standard procedure or guidelines. - **Unobserved:** Step, task, or skill not performed by the learner during assessment by the clinical assessor. - **Not applicable:** Step, task, or skill not applicable |
| --- |

Employee's name: _______________________________

Questionnaire number: ______________________

| **Checked by** | **Name** | **Date** | **Signature** |
| --- | --- | --- | --- |
| Interviewer: |  |  |  |
| Verified in the field by: |  |  |  |
| Verified at the office by: |  |  |  |
| Data recorded: |  |  |  |

| 1. Date of visit |  |
| --- | --- |
| 2. Region  ***(circle the corresponding code)*** | Boucle du Mouhoun Region ...........................1  Hauts-Bassins Region …………………………………..2  Center Ouest Region ......................................3 |
| 3. Health district  ***(circle the corresponding code)*** | Do ........................................................  Dafra ........................................................2  Dedougou ........................................................3  Boromo..................................................4  Leo ........................................................5  Sapouy ........................................................6 |
| 4. Health Facility  ***Enter the name of the health facility*** | ________________________________________________________ |
| 5. Type of Health Facility  ***(circle the corresponding code)*** | CHU (University Hospital) ......................... ...1  CHR (Regional Hospital) ...............................2  District Hospital ....................................3  CM (Medical Center) .....................................4  CSPS (Specialist Center) ...............................5 |

| **Checklist for counseling and clinical skills for implant removal** | | |
| --- | --- | --- |
| **Task** | **Assessment** | |
| **Pre-removal counselling** | | |
| 1. Greets customers with friendliness and respect. |  | |
| 1. Listens carefully to the client's response as to the reason for implant removal to determine whether she wishes to use another method, hopes to become pregnant, or wants to replace her implant. |  | |
| 1. Confirms the customer's intentions. Provides FP counseling, as needed. |  | |
| 1. Describes the removal procedure and what to expect. |  | |
| 1. If she is considering another implant, discusses where it will be inserted. |  | |
| 1. Checks that the client is not allergic to the topical antiseptic or local anesthetic available. |  | |
| **Tasks performed competently** |  | |
| **Preparation** | | |
| 1. Ensures that sterile instruments and other supplies required for removal are available. |  | |
| 1. Ensures that a new implant is available if you insert a new implant. |  | |
| 1. Checks that the client has washed her arm with soap and rinsed it. |  | |
| 1. Explains to the client what will be done and encourages her to ask questions. |  | |
| 1. Positions the woman's arm and places a clean, dry cloth under her arm. |  | |
| 1. Palpates the rod(s) to determine the point of removal. |  | |
| 1. With a permanent marker, make a mark on the client's arm where the tip of the rod(s) is/are palpated. |  | |
| **Tasks performed competently** |  | |
| **Pre-removal tasks** | | |
| 1. Washes hands thoroughly and air-drys or drys with a clean towel. |  | |
| 1. Wears sterile gloves on both hands. |  | |
| 1. Arranges instruments and supplies. |  | |
| 1. Prepares the insertion site by wiping twice with an antiseptic solution. |  | |
| 1. Injects a small amount of local anesthetic (1% without epinephrine) at the incision site and under the tip of the rod(s). |  | |
| 1. Checks the effect of the anesthesia before making the incision. |  | |
| **Tasks performed competently** |  | |
| **Removal** | | |
| 1. Presses on the proximal end of the implant to immobilize it (a bulge indicating the distal end of the implant may appear). |  | |
| 1. Makes a small incision (2 mm) under the end of the stick. |  | |
| 1. Pushes the end of the stick towards the incision, then removes it. |  |  |
| 1. Grasps the end of the stick with curved forceps (Crile or mosquito forceps). |  |  |
| 1. Removes the sheath of fibrous tissue covering the tip of the stick with a sterile compress (or with the back of the scalpel blade). |  |  |
| 1. Grasps the exposed end of the stick with the second pair of forceps, carefully removes and checks that the stick is intact before placing it in a bowl containing 0.5% chlorine solution for decontamination. |  |  |
| 1. Ensures that the complete stick is removed and shows it to the client. |  |  |
| 1. For a two-rod implant, repeats steps 22 to 26. |  | |
| **Tasks performed competently** |  | |
| **Reinsertion of the implant (one or two rods)** | | |
| *The rod(s) of the new implant can be inserted in the same location as the implant that was recently removed (if the woman has chosen to have a new implant).* |  | |
| 1. Applies an additional local anesthetic by infiltrating 4cc of 1% lidocaine at the site of the implant(s) previously removed. |  | |
| 1. Waits 1 to 2 minutes for the anesthetic to take effect. |  | |
| 1. Inserts the single- or double-rod implant according to the insertion instructions  (including post-insertion steps and post-insertion counseling). |  | |
| **Tasks performed competently** |  | |
| **Post-removal tasks** | | |
| 1. Wipes the client's skin with betadine or another antiseptic. |  | |
| 1. Brings the incision edges together for hemostasis then covers with a dressing or tape on a sterile compress (2x2cm). |  | |
| 1. Applies a pressure bandage with moderate pressure. |  | |
| 1. Before removing gloves, removes material by:  - Placing the used needle (without cap) and trocar in a sharps container, and by - Placing the waste in an airtight container or plastic bag, and by - Placing the forceps in a container containing a 0.5% chlorine solution. |  | |
| 1. Removes his/her gloves by reversing them and throws them in a sealed container or a plastic bag. |  | |
| 1. Washes and dries hands thoroughly. |  | |
| 1. Fills out the client's file. |  | |
| **Tasks performed competently** |  | |
| **Post-removal counselling** | | |
| 1. Gives the customer instructions on how to care for the wound, then schedules the next visit, if necessary. |  | |
| 1. Discusses what to do in the event of a problem and answers any questions. |  | |
| 1. Advises clients on new contraceptive methods and provides them if they wish. |  | |
| 1. Keeps the client under observation for at least 15 minutes before letting her go. |  | |
| **Tasks performed competently** |  | |

Clinical skills achieved: On models □ On clients □

**Signature of clinical assessor: Date:**

**Checklist: Clinical skills: removal of difficult-to-extract implants**

Modified U technique for implant removal

| **Checklist for counseling and clinical skills: implant difficult to extract** | | |
| --- | --- | --- |
| **Task** | **Assessment** | |
| **Pre-removal counselling** | | |
| 1. Greets customers with friendliness and respect. |  | |
| 1. Listens carefully to the client's response as to the reason for implant removal to determine whether she wishes to use another method, hopes to become pregnant, or wants to replace her implant. |  | |
| 1. Confirms the customer's intentions. Provides FP counseling, as needed. |  | |
| 1. Assesses the visibility, location and position of the rod(s) by palpation. |  | |
| 1. If the rod(s) is (are) not palpable, consults X-ray or ultrasound reports to identify the position if available. |  | |
| 1. If not, prescribes an X-ray or ultrasound. |  | |
| 1. Describes the removal procedure and what to expect. |  | |
| 1. If she's considering another implant, discusses where it will be inserted. |  | |
| 1. Checks that the client is not allergic to the topical antiseptic or local anesthetic available. |  | |
| **Tasks performed competently** |  | |
| **Removing the rod(s)** | | |
| Preparation | | |
| 1. Ensures that sterile instruments and other supplies required for removal are available. |  | |
| 1. Ensures that a new implant is available if the client requires a new implant. |  | |
| 1. Checks that the client has washed her arm with soap and rinsed it. |  | |
| 1. Explains to the client what will be done and encourages her to ask questions. |  | |
| 1. Positions the woman's arm and place a clean, dry cloth under her arm. |  | |
| 1. Notes the location of the rods by deep palpation or by consulting the X-ray or ultrasound. |  | |
| 1. With a permanent marker, makes a mark on the client's arm where the tip of the rod(s) is/are palpated. |  | |
| **Tasks performed competently** |  | |
| **Pre-removal tasks** | | |
| 1. Washes hands thoroughly and drys them with a single-use towel or air dryer. |  | |
| 1. Puts sterile gloves on both hands, using the "no-touch" technique. |  | |
| 1. Arranges instruments including modified ring pliers and supplies. |  | |
| 1. Prepares the insertion site by wiping twice with an antiseptic solution. |  | |
| 1. Lifts the skin and injects 1.5 ml of 1% local anaesthetic at the incision site at the tip of the rod(s) located by palpation or observed on X-ray or ultrasound. |  | |
| 1. Checks the effect of the anesthesia before making the incision. |  | |
| **Tasks performed competently** |  | |
| **Removing the rod(s)** | | |
| 1. Arranges instruments and supplies on sterile linen. |  | |
| 1. Makes a 4 mm longitudinal incision at the most superficial end of the rod(s). Then, carefully and gently dissects the tissue with mosquito forceps until the implant can be felt. |  | |
| 1. While pressing firmly on the stick with the index finger of the non-dominant hand, inserts the modified ring forceps gently downwards into the incision, until the rod is located. |  |  |
| 1. Passes the modified ring pliers under the rod, while pressing on the rodwith the index finger of the non-dominant hand. |  |  |
| 1. Tilts the modified ring clamp, opens it, and grasps the rod while the index finger pushes it into the ring. |  |  |
| 1. Grasps the rod body and push it towards the incision using the right-hand mosquito forceps to remove the fibrous tissue sheath covering the rod tip. |  |  |
| 1. Using the curved mosquito pliers, grasps the stick and releases the modified ring pliers. |  |  |
| 1. Lifts the implant towards the middle, pulling it into a U-shape. Cleans the surrounding tissue and releases the rod. |  |  |
| 1. Makes sure to have removed the whole rod and shows it to the client. |  |  |
| 1. For a two-rod implant, repeats steps 25-31. |  | |
| **Tasks performed competently** |  | |
| **Reinsertion of implant (single- or double-rod)** | | |
| *The rod(s) of the new implant can be inserted in the same location as the implant just removed, if the removal was not too difficult* | | |
| 1. Applies an additional local anesthetic by injecting 1% lidocaine at the site of the implant(s) previously removed. Administers 1ml per stick. |  | |
| 1. Waits 1 to 2 minutes for the anesthetic to take effect |  | |
| 1. Inserts single- or double-rod implant according to insertion instructions  (including post-insertion steps and post-insertion counseling) |  | |
| **Tasks performed competently** |  | |
| **Post-removal tasks** | | |
| 1. Wipes the client's skin with a swab soaked with the indicated antiseptic. |  | |
| 1. Brings the incision edges together and closes them with surgical plaster, then covers with dressing or tape on a sterile compress (2x2). |  | |
| 1. Applies a pressure bandage with moderate pressure. |  | |
| 1. Before removing gloves, removes material by:  - Placing the used needle (without cap), trocar, and surgical blade in a sharps container, and by - Placing the waste in an airtight container or plastic bag, and by - Places the forceps in a container containing a 0.5% chlorine solution. |  | |
| 1. Removes his/her gloves by reversing them and throws them in a sealed container or a plastic bag. |  | |
| 1. Washes hands thoroughly and dries with a single-use towel or air dryer. |  | |
| 1. Completes client record and daily activity log. |  | |
| **Tasks performed competently** |  | |
| **Post-removal counselling** | | |
| 1. Gives instructions to the client on how to care for the wound and when to return to the Health Center in the following cases:  - Heavy bleeding - Pus or redness at the incision site - Another concerns. |  | |
| 1. Discusses what to do in the event of a problem and answers any questions that arise. |  | |
| 1. Advises the client on a new contraceptive method if she has not accepted the implant and provides it if she so wishes. |  | |
| 1. Keeps the client under observation for at least 15 minutes before letting her go. |  | |
| **Tasks performed competently** |  | |

Clinical skills achieved: On models □ On clients □

**Signature of clinical assessor: Date:**
