# Supplementary material for: Mobilizing stakeholders for implant removals in Burkina Faso using landscape assessment data
Source: BMC Womens Health. 2024 May 20;24:301. doi: 10.1186/s12905-024-03121-z (PMC11104007; doi:10.1186/s12905-024-03121-z)
Supplement: Supplementary file 2 — Supplementary Material 2. [file 12905_2024_3121_MOESM2_ESM.docx]

**Principal investigator:** Yacouba Ouedraogo

**Study title:** Situation analysis on the availability and quality of contraceptive implant removal services in Burkina Faso in 2018.

**Date:** September 20, 2018

**Tool 2: Interview with the provider of the Health Facility observed during the contraceptive implant removal service**

Name of the interview organizer: _______________________________

Questionnaire number: ______________________

| **Checked by** | **Name** | **Date** | **Signature** |
| --- | --- | --- | --- |
| Interviewer: |  |  |  |
| Verified in the field by: |  |  |  |
| Verified at the office by: |  |  |  |
| Data recorded: |  |  |  |

| 1. Date of visit |  |
| --- | --- |
| 2. Region |  |
| 3. Health district |  |
| 4. Health Facility |  |
| 5. Type of Health Facility  (circle 1) | CHU (University Hospital) =1  CHR (Regional Hospital) =2 District Hospital=3  CM (Medical Center) =4 CSPS (Specialized Center) =5 |

**MLDA/contraceptive implant service provider capabilities**

| 6. For follow-up procedures, please indicate the type of training received (check all that apply): | | | | | | |
| --- | --- | --- | --- | --- | --- | --- |
| Type of service | Training outside the service provider's premises | Training at the service provider's work site | Training during pre-service school studies | On-the-job formal training/continuing education | Informal on-the-job training | Did not receive any training for this procedure |
| Implant insertion |  |  |  |  |  |  |
| Implant removal |  |  |  |  |  |  |

**Note:** *Off-site:* outside the service organization, *On-site:* within the service organization, *Structured:* with a defined, well-organized curriculum with practical and didactic sessions, *Unstructured:* learning by observing a qualified provider offer the service, not a well-defined curriculum.

7. If you have had training on contraceptive implants, when did the most recent session occur?

Month _________________ Year _______________________

8. During your training,

a. Did you encounter a shortage of clients for contraceptive implant removal during training?

| i. Contraceptive implants | Yes ☐ No ☐ Did not have a client☐ |
| --- | --- |

b. If so, how was the lack of clients managed?
__________________________________________________________________________________
__________________________________________________________________________________
__________________________________________________________________________________
__________________________________________________________________________________

| 9. **Provider's confidence in service methods**  *On a scale of 1 to 5 (1 is "*not confident*", 2* is "somewhat confident", *3 is "*moderately confident*", 4 is "*confident*" and 5 is "*very confident*")*.  *To what extent do you carry out the following procedures?* | | |
| --- | --- | --- |
| a. Contraceptive implant insertion | 1 2 3 4 5 I do not provide this service | |
| b. Contraceptive implant removal | 1 2 3 4 5 I do not provide this service | |
| 10. Have you ever removed a contraceptive implant from a client? | Yes ☐ No ☐ | |
| 11. Have you ever had problems removing contraceptive implants? | Yes ☐ No ☐ | |
| a. If yes, which?  (check all that apply) | 1. Deeply inserted ☐ 2. Excessive bleeding ☐  3. Missing pieces ☐  4. Insufficient/missing instruments/equipment☐ 5. Insufficient/missing consumables ☐  6. Heavy workload ☐ 7. Ability to pay (Cost) ☐ 8. Lack of knowledge ☐ 9. Other (please specify) ___________________________________________ ____________________________________________ | |
| 12. What are the main challenges you face in using the equipment? | a) Electrical failure b) Too much work c) Defective autoclave d) Other (please specify) ______________________________________ | |
| 13. Have you ever had to remove a difficult contraceptive implant because it was placed too deeply inside a client? | Yes ☐ No ☐ | |
| 1. In the last 3 months, have you encountered any difficult removals? | Yes ☐ No ☐ | |
| 1. If a customer comes in to undergo a difficult removal or deep insertion, etc., what course of action do you take? Please list all cases in which a client could be referred, if applicable. | *Please list all cases where a client could be referred; if applicable.* ________________________________________________________________________________ ________________________________________________________________________________ ________________________________________________________________________________ ________________________________________________________________________________ | |
| 1. Have you come across a case where a client went to the health center to have a contraceptive implant removed, but was unable to have it removed? ? | | Yes ☐  No ☐ |
| a. If yes, please state the reason (circle all that apply) | | 1) Facility not open 2) Qualified provider not available 3) Provider attempted but was unable to remove contraceptive implant 4) Materials and equipment for removal not available. 5) Untreated (sterile) equipment 6) Cost of removal service 7) Provider advised against removal 8) Other (please specify) : _________________________ |
| 1. In the past 3 months, have you met a client who was unable to have her contraceptive implant removed because she was unable to pay for the service? | | Yes ☐  No ☐ |
| 1. Does your facility have documents to guide providers on contraceptive implant removal? | | Yes ☐  No ☐ |
| 1. If yes to question 19, what are these documents? (Please list the documents) | | ____________________________________________________________________________________________________________________________________________________________________________________________________________________________________________________________________________________________________________________ |
| 1. With regard to data on the removal of long-acting contraceptive methods, how are contraceptive implant removals recorded in this facility? | | ____________________________________________________________________________________________________________________________________________________________________________________________________________________________ |
| 1. Are there any difficulties to report in the removal process? | | Yes ☐ No ☐ |
| 1. If yes to question 22, what are these difficulties? | | List the difficulties encountered:  ____________________________________________________________________________________________________________________________________________________________________________________________________________________________________________________________________________________________________________________ |
| 1. Has this health facility received reproductive health support supervision in the last 3 months? | | Yes ☐  No ☐ |
| 1. According to the data in your health facility, what are the most preferred contraceptive methods in this community? (Give 3) | | ____________________________________________________________________________________________________________________________________ |
| 1. Why do you think members of this community prefer these methods? (Look for details in the answers, especially from the client's point of view) | | 1) Method-related expenses  2) Duration of effectiveness  3) Discretion  4) Traditional religion/beliefs  5) Ease of use  6) Geographical accessibility  7) Frequency of follow-up appointments linked to the method  8) Other (please specify)  ____________________________________________ |
| 1. What do clients say about their experiences with contraceptive implants? (Look for details) | | ________________________________________________________________________________________________________________________________________________________________________________ |
| 1. On the positive side? | | ____________________________________________________________________________________________________________________________________________________________________________________________________________________________________________________________________________________________________________________ |
| 1. On the negative side? | | ____________________________________________________________________________________________________________________________________________________________________________________________________________________________ |
| 1. Based on your experience, please share reflections/suggestions on what might be the most effective strategies for strengthening implant removal services in this Health Facility. | | ____________________________________________ ____________________________________________ ____________________________________________  ____________________________________________________________________________________________________________________________________ |

|  |
| --- |
|  |
| 1. Do you have any other comments or questions to share with me? (Specify if any) |

______________________________________________________________________________________________________________________________________________________________________________________________________________________________________________________________________________________________________________________________________________________________________________________________________________________________________________________________________________________________________________________________________________________________________________________________________________________________________________________________________________________________________________________________________________________________________________________________________________________________________________________________________________________________________________________________________
