# Supplementary material for: Mobilizing stakeholders for implant removals in Burkina Faso using landscape assessment data
Source: BMC Womens Health. 2024 May 20;24:301. doi: 10.1186/s12905-024-03121-z (PMC11104007; doi:10.1186/s12905-024-03121-z)
Supplement: Supplementary file 3 — Supplementary Material 3. [file 12905_2024_3121_MOESM3_ESM.docx]

**Principal investigator:** Yacouba Ouedraogo

**Study title:** Situation analysis on the availability and quality of contraceptive implant removal services in Burkina Faso in 2018.

**Date:** September 20, 2018

**Tool 3: Evaluation of contraceptive implant removal services with a family planning focal point in the Health Facility**

*Evaluating the Health Facility with the manager.*

The aim of this health facility analysis is to gather key information on the state of contraceptive implant (hereinafter "implant") removal services and then share this information with the Ministry of Health through FP (Family Planning). Specifically:

- What challenges do health facilities and FP providers face in offering implant removal services?
- What happens at your facility when a client comes in for implant removal and the service isn't available?
- NB: *(*For hospitals, ask the head of the Maternal Health Department, and for health centers, ask the provider in charge of family planning*)*

Name of the interview organizer: _______________________________

Questionnaire number: ______________________

| **Checked by** | **Name** | **Date** | **Signature** |
| --- | --- | --- | --- |
| Interviewer: |  |  |  |
| Verified in the field by: |  |  |  |
| Verified at the office by: |  |  |  |
| Data recorded: |  |  |  |

1. **General information**

| 1. Visit date |  |
| --- | --- |
| 2. Region |  |
| 3. Health district |  |
| 4. Health Facility |  |
| 5. Type of Health Facility  (circle 1) | CHU (University Hospital)=1  CHR (Regional Hospital =2 District Hospital = CM (Medical Center)=4  CSPS (Specialized Center)=5 |
| 6. Name of service provider interviewed (Optional) | __________________________________  Tel No. ___________________ |
| 7. Responsability | Facility in charge □ MCH (Maternal Child Health Supervisor) in charge □  Other (please specify) |
| 8a. Qualification  (circle 1) | Doctor = 1 Nurse = 2 Midwife= 3  Certified midwife = 4 Assistant midwife = 5  Itinerant healthcare worker = 6 |
| 8b. Number of years as service provider |  |
| 9. What is the number of providers per qualification in this health facility?  *Enter the number directly in the answer field in front of each agent category.* | Gynecologists /___/  Doctors /___/  Surgical nurses /___/  Nurses /___/  Midwives /___/  Assistant midwives /___/  Certified midwife  Itinerant healthcare worker /___/ |

1. **Service provision for long-acting methods.**
   1. How many providers are able to offer implants in this training course?
   2. For which type of implant have they been trained (check):

Classic implant __

NEXplanon (or NEX implant) __

Jadelle __

- 1. How many providers provide follow-up appointments in the Health Facility and how often?

| **Type of services** | Inserting implants | Removing implants | Removal of difficult/and or non-palpable implants |
| --- | --- | --- | --- |
| **Training service providers** |  |  |  |

- 1. How often are the services available in the Health Facility monitored? (Give answers for each service)

| **Type of services** | Inserting implants | Removing implants | Removal of difficult/and or non-palpable implants |
| --- | --- | --- | --- |
| 5 d/wk 8AM -5PM |  |  |  |
| 7 d/wk 8 AM - 5 PM |  |  |  |
| 24/7 |  |  |  |
| Specific weekdays |  |  |  |

- 1. Does this facility have a quality improvement system/team?
     1. Yes
     2. No
     3. If so, how is the team organized and how does it operate?

________________________________________________________________________________________________________________________________________________________________________________________________________________________________________________________________________________________________________________________________________________________________________________________________________________________________________________________________________________________________________________________________________________

- 1. If so, how often is it organized?
     1. Weekly
     2. Monthly
     3. Quarterly
     4. Other (please specify) -------------------------------------------------------------------
  2. Does this Health Facility have mentors for long-acting contraceptive methods?
     1. Yes
     2. No
  3. Does structured LARC (Long-Acting Reversible Contraceptive) mentoring exist in this Health Facility? (a defined, clear curriculum with learning objectives and a timetable for mentees, a defined mentoring action plan, mentor-mentee etc.).
     1. Yes
     2. No
  4. If so, how often does structural mentoring take place?
     1. Never
     2. Rarely
     3. Often
     4. Always
  5. Do you have funding to support FP activities?
     1. Yes
     2. No
  6. If yes, what is the source of funding?
     1. The Health Facility's own funds
     2. Government
     3. Partners/NGOs (Specify)........................................
     4. Other (Please specify)………………………………………………….
  7. *What does the client pay for the service?* (*NB: put "0" if the customer pays nothing)*

| **Service** | **Registration /Consultation** | **Service Manager** | **Total** |
| --- | --- | --- | --- |
| Implant insertion |  |  |  |
| Implant removal |  |  |  |

- 1. Does this Health Facility conduct community FP activities?
     1. Yes
     2. No

*If yes, are they functional?*

- 1. When was the last time you had an awareness campaign?

Month ___________ Year ____________

- 1. Who supports you in organizing awareness campaigns?
     1. The Health Facility itself
     2. Partner/NGO (specify)
     3. Ministry of Health (without NGO/partner)
  2. Does the Health Facility provide any follow-up for community activities/campaigns?

| **Type of service** | **Yes** | **No** |
| --- | --- | --- |
| Implant insertion |  |  |
| Implant removal |  |  |
| Other (If yes, which - pills, injectables, condoms, etc.) |  |  |

- 1. Does the Health Facility conduct on-site campaigns?
     1. Yes
     2. No
         If yes, when was the last time you had an on-site campaign month ___________ year ____________
         What is the source of funding for on-site campaigns?

| i. The Health Facility's own resources  ii. Partners/NGOs (specify) | □  □ _____________________ |
| --- | --- |

- 1. Are the following services provided during "Health Facility Campaigns"?

| **Type of service** | **Yes** | **No** |
| --- | --- | --- |
| Implant insertion |  |  |
| Implant removal |  |  |
| Other (If yes, which - IUD, pills, injectables, condoms, etc.) |  |  |

- 1. Do you have the capabilities to offer difficult implant removal even for non-palpable implants?
     1. Yes
     2. No
        If so, who removes difficult implants?
        1. Physician OBS/GYN ---
        2. Doctors
        3. Nurses
        4. Other (please specify) _________________________________________
  2. Have you set up a referral mechanism for the difficult removal of implants, including non-palpable ones?
     1. Yes
     2. No

If so, to which health facility do you usually refer?
Name of the facility: _________________________________________
- Type:

- CHU (University Hospital) ☐
- CHR (Regional Hospital) ☐
- District Hospital ☐
- CM (Medical Center) ☐
- CSPS (Specialized Center) ☐
- Other to be specified ______________________________
  1. Based on your experience, please share thoughts/suggestions on what might be the most effective strategies for strengthening implant removal services in this Health Facility.
     __________________________________________________________________
     __________________________________________________________________
     __________________________________________________________________
  2. Does this Health Facility or Department have the following equipment and materials**? Indicate Yes or no**

Checklist of equipment and consumables available at the Health Facility for implant insertion and removal

| **Item** | **Available?** | **Observed?** | **Appropriate?** |
| --- | --- | --- | --- |
| **Implant insertion/removal** | | | |
| Kidney dishes / Emesis basin |  |  |  |
| Gallipot cupula |  |  |  |
| Halstead mosquito forceps, right |  |  |  |
| Curved mosquito forceps |  |  |  |
| Modified vasectomy right vascular clamp |  |  |  |
| Scalpel blades |  |  |  |
| Scalpel holder |  |  |  |
| Clamps |  |  |  |
| Lidocaine without epinephrine 1% (lidocaïne 2%) |  |  |  |
| 5cc syringe |  |  |  |
| Water for injection preparation |  |  |  |
| Sterile compresses |  |  |  |
| Sterile bandage/adhesive bandage |  |  |  |
| Sterile gloves |  |  |  |
| Iodized polyvidone |  |  |  |
| For difficult removals |  |  |  |
| Ultrasound |  |  |  |
| Radiography/x-ray machine(s) |  |  |  |
| **Other equipment** | | | |
| Functional autoclave |  |  |  |
| Gynecological table |  |  |  |
| Light source |  |  |  |
| **Infection prevention** | | | |
| Running water |  |  |  |
| Decontamination receptacles |  |  |  |
| Safety boxes |  |  |  |
| Soap |  |  |  |
| Chlorine water |  |  |  |

Comments on equipment and material:
__________________________________________________________________________________
__________________________________________________________________________________
__________________________________________________________________________________
__________________________________________________________________________________

- 1. Physically count the number of clients who have benefited from the following services over the past three months in the daily activity register.

NB: Source: register of family planning activities; total includes campaign outside the Health Facility + campaign within the Health Facility+ statistics service of the day)

| Method | May 2018 | | | June 2018 | | | July 2018 | | |
| --- | --- | --- | --- | --- | --- | --- | --- | --- | --- |
| Point of service | FS routine data | Community campaign | Total | FS routine data | Community campaign | Total | FS routine data | Community campaign | Total |
| Implant insertion |  |  |  |  |  |  |  |  |  |
| Implant removal |  |  |  |  |  |  |  |  |  |
