# Supplementary material for: Mobilizing stakeholders for implant removals in Burkina Faso using landscape assessment data
Source: BMC Womens Health. 2024 May 20;24:301. doi: 10.1186/s12905-024-03121-z (PMC11104007; doi:10.1186/s12905-024-03121-z)
Supplement: Supplementary file 4 — Supplementary Material 4. [file 12905_2024_3121_MOESM4_ESM.docx]

**Principal investigator:** Yacouba Ouedraogo

**Study title:** Situation analysis on the availability and quality of contraceptive implant removal services in Burkina Faso in 2018.

**Date:** September 20, 2018

## Tool 4: Interview guide for managers/decision-makers

**Introduction**

The aim of this health facility analysis is to gather key information on the state of contraceptive implant (hereinafter: "implant") removal services and then share this information with the Ministry of Health through FP (Family Planning). Specifically:

- What challenges do health facilities and FP providers face in offering implant removal services?
- What happens in your facility when a client comes in for implant removal and this service isn't available?
- NB: *(*For hospitals, ask the head of the Maternal Health Department, and for health centers, ask the provider in charge of family planning*)*

We'd like to hear your views on family planning services, and in particular on implant removal. Given your responsibility for the healthcare system, we want to talk to you about implant removal.

In fact, the Ministry of Health, in collaboration with Jhpiego with funding from Bill & Melinda Gates, is seeking to gain a better understanding of the implant removal situation in the country, with a view of improving implant removal offers. So, we would like to talk to you about this subject. Your answers will help us understand the situation and guide our actions regarding implant removal in Burkina Faso.

**Interview start time: ________:________**

Employee's name: _______________________________

Questionnaire number: ______________________

| **Checked by** | **Name** | **Date** | **Signature** |
| --- | --- | --- | --- |
| Interviewer: |  |  |  |
| Verified in the field by: |  |  |  |
| Verified at the office by: |  |  |  |
| Data recorded: |  |  |  |

**1. Information about the respondent:**

| 1. Date of visit |  |
| --- | --- |
| 1. Location |  |
| 1. Profession |  |
| 1. Gender (M/F) |  |
| 1. Organization/Institution |  |
| 1. Unit/services |  |
| 1. Length of time in position (years and months) |  |

## General information

What role does your department/program/division/organization play in family planning in Burkina Faso?

_______________________________________________________________________________________________________________________________________________________________________________________________________________________________________________________________________________________________________________________________________________________________________________________

## Availability of documents

1. As part of the situational analysis of implant removal, we have a literature review component. To make sure we've taken into account all the texts and standards governing the field of implant removal, we'd like to have a list of the documents.

_____________________________________________________________________________________________________________________________________________________________________________________________________________________________________________________________________________________________________________________________________________________________________________________________________________________________________________________________________________________________________________________________________________

1. What is your assessment of the texts, documents, and standards governing family planning in general? What about implant insertion and removal in particular?

i) Policy document__________________________________________________________________________________________________________________________________________________________________________________________________________________________ ii)Standards_________________________________________________________________________________________________________________________________________________________________________________________________________________________ iii)Training______________________________________________________________________________________________________________________________________________________________________________________________________________________ iv)Guides and protocols _________________________________________________________________________________________________________________________________________________________________________________________________________________________________ v)Other______________________________________________________________________

1. What documents do you know of regarding the insertion/removal of implants?

____________________________________________________________________________________________________________________________________________________________________

d) Are these texts and documents available in the field, and at what level can they be found? ____________________________________________________________________________________________________________________________________________________________________

e) How are these texts and documents used by service providers?

____________________________________________________

f) What strategy is used in the field to ensure that providers use **documents?** ___________________________________________________________________________________________________________________________________________________________________________________________________________________________________________

## Training

1. How are skills and education acquired by implant removal service providers? ____________________________________________________________________________________________________________________________________________________________________

b) In the organization that provides implant insertion/removal training courses, is there a specific module on removal (simple, difficult, or non-palpable implant removal) with verification lists? Can you share them with us?

______________________________________________________________________________________________________________________________________________________________________________________________________________________________________________________

c) What activities are carried out during pre-service training to help students acquire skills? ______________________________________________________________________________________________________________________________________________________________________________________________________________________________________________________

d) In your opinion, do new graduates from pre-service training schools have the necessary skills to offer implant removal services? Please explain. ______________________________________________________________________________________________________________________________________________________________________________________________________________________________________________________

e) How and at what rate are service providers already in the field updated on processes? Who provides these updates?

______________________________________________________________________________________________________________________________________________________________________________________________________________________________________________________

f) When was large-scale training carried out for providers in implant insertion and removal? Who organized and financed this training, and what profiles were involved?

___________________________________________________________________________________________________________________________________________________________________

## Service performance and data management

## What strategies do you use to regularly assess the skills of implant removal service providers in the field?

______________________________________________________________________________________________________________________________________________________________________________________________________________________________________________________

1. What types of health facilities are authorized to provide implant removal services to the public?

______________________________________________________________________________________________________________________________________________________________________________________________________________________________________________________

1. Describe the system implemented by the program to manage difficult implant removals (algorithm and reference system)?

______________________________________________________________________________________________________________________________________________________________________________________________________________________________________________________

d) In your opinion, what are the current challenges and needs in terms of implant removal?

i. easy/simple removal? ____________________________________________________________________________________________________________________________________________________________________

ii. difficult removal?

____________________________________________________________________________________________________________________________________________________________________iii. Others?

____________________________________________________________________________________________________________________________________________________________________

1. In your opinion, what are the biggest barriers to the provision of implant removal services, and why?

______________________________________________________________________________________________________________________________________________________________________________________________________________________________________________________

f) How is data collected, processed, and used at peripheral, intermediate, and central levels? ______________________________________________________________________________________________________________________________________________________________________________________________________________________________________________________

______________________________________________________________________________________________________________________________________________________________________________________________________________________________________________________

## Equipment

1. How are health facilities equipped to remove implants? ____________________________________________________
2. Who estimates needs, and how often? ____________________________________________________
3. Is there a standard list of equipment for implant removal? ___________

d.) What types of health facilities are available? ____________________________________________________________________________________________________________________________________________________________________

e.) How often are health facilities equipped?

____________________________________________________

1. Is there a monitoring system for implant removal equipment?

____________________________________________________

1. Do you have any idea of the coverage (as far as you are concerned) of health facilities in terms of availability of equipment?

____________________________________________________________________________________________________________________________________________________________________

## Problems and difficulties

a) What is your general assessment of supply and demand for implant removal (in your country, region, or district)? ______________________________________________________________________________________________________________________________________________________________________________________________________________________________________________________

b) Do you think there are any problems or difficulties with the implant removal service? If yes, which?

i. If cases of difficult implant removals have been reported, what area is involved? ______________________________________________________________________________________________________________________________________________________________________________________________________________________________________________________

c) What solutions do you have to suggest to improve implant services? ______________________________________________________________________________________________________________________________________________________________________________________________________________________________________________________

## Outlook

a) What can be done to ensure the quality of the implant removal services available and accessible?

______________________________________________________________________________________________________________________________________________________________________________________________________________________________________________________

b) What contribution can your organization/institution make to support the implementation of quality services for implant removal?

______________________________________________________________________________________________________________________________________________________________________________________________________________________________________________________

c) If assistance were needed to improve implant removal services, what would you suggest?

_________________________________________________________________________________________________________________________________________________________________________________________________________________________________________________________________________________________________________________________________________________________________________________________________________________________

1. Is there anything else you can tell us about implant removal that hasn't been covered?

____________________________________________________________________________________________________________________________________________________________________

**THANK YOU FOR YOUR COLLABORATION**
